# Supplementary material for: Migration intentions and their impact on healthcare workers in a Lebanese public university hospital amid crises: A mixed-method study
Source: PLoS One. 2026 Feb 4;21(2):e0341426. doi: 10.1371/journal.pone.0341426 (PMC12871958; doi:10.1371/journal.pone.0341426)
Supplement: S1 Appendix — (DOCX) [file pone.0341426.s001.docx]

**S1 Appendix Interview Topic Guide**

**Interview Questions and Probes**

**Introduction**

Global shortages of healthcare workers represent a critical issue that threatens the sustainability of health systems worldwide. This global movement phenomenon of skilled healthcare workers has a significant impact on healthcare quality in home countries because of resource loss, imbalances in workforce distribution, and healthcare provision. The culmination of multiple crises in Lebanon has exacerbated brain drain, which is occurring today at an alarming speed.

**Q1: General Idea of Healthcare Workers' Migration**

- **Main Question:** Can you give us a general idea of healthcare workers who migrated from your department at Rafik Hariri University Hospital (RHUH) in the last period?
  - **Probe 1:** Can you give us an estimation of the number of healthcare workers who migrated from your department in the last period?
  - **Probe 2:** In general, where did most healthcare workers migrate to? (MENA region or outside MENA)

**Q2: Reasons for Migration**

- **Main Question:** In your opinion, what do you think are the reasons for healthcare workers' decision to migrate?

**Q3: Impact of Migration**

- **Main Question:** What is the impact of healthcare workers’ migration at the hospital level?
  - **Probe 1:** What is the impact of healthcare workers' migration at the staff level? (Workload for the remaining staff)
  - **Probe 2:** What is the impact of healthcare workers’ migration at the healthcare service level? (Service provision, quality of care)
  - **Probe 3:** What is the impact of healthcare workers' migration at the management level?

**Q4: Current Retention Strategies**

- **Main Question:** What actions have you already implemented to retain qualified healthcare workers at RHUH?

**Q5: Future Retention Strategies**

- **Main Question:** In your opinion, what actions could be taken in the future to retain healthcare workers at RHUH?
  - **Probe 1:** Can you name any technical strategies?
  - **Probe 2:** Can you name any financial strategies?
  - **Probe 3:** Can you name any communication strategies?
  - **Probe 4:** What about sustainable strategies?

**Q6: Additional concerns and questions**

- **Main Question:** Are there any additional concerns or issues you would like to talk about? Do you have any questions for us?
