# Supplementary material for: Migration intentions and their impact on healthcare workers in a Lebanese public university hospital amid crises: A mixed-method study
Source: PLoS One. 2026 Feb 4;21(2):e0341426. doi: 10.1371/journal.pone.0341426 (PMC12871958; doi:10.1371/journal.pone.0341426)
Supplement: S1 Table — (DOCX) [file pone.0341426.s002.docx]

**S1 Table Summary of Staff Suggestions for Improving Retention at RHUH**

| **Category** | **Subcategory** | **Suggestion** |
| --- | --- | --- |
| **Salary and Financial** | Salary increase | Significant salary raises |
|  |  | Adjustment to match economic situation |
|  |  | Payment in fresh dollars or equivalent |
|  |  | Increase in overtime value |
|  |  | Pay 50% of salary in dollars, 50% in LBP |
|  |  | Fresh dollar portion of salary (20% or more) |
|  |  | Economic and social safety |
| **Promotion and development** | Promotion opportunities | Promotion based on certificate and competence |
|  |  | Promotion according to qualifications |
|  | Professional Development | Participation in training courses |
|  |  | Continuous training on new skills |
|  |  | Training sessions outside Lebanon |
|  |  | Contract with universities for staff education and training |
|  |  | Coverage of training costs and attendance |
|  |  | Development and updating of the Human Resources department |
| **Work Environment** | Adequate Resources | Ensuring sufficient staff in departments |
|  |  | Providing necessary equipment and resources |
|  |  | Ensuring cleanliness standards, especially in operations |
|  | Comfortable and Safe Conditions | Comfortable and safe working environment |
|  |  | Rest breaks during duty |
|  |  | Decrease in working hours |
|  |  | Reducing patient-to-nurse ratio |
| **Rewards and Job Security** | Reward and Punishment Mechanism | Implementation of reward and punishment mechanism |
|  |  | Respect and good treatment of employees |
|  |  | Employee rights recognition |
|  | Job Security | Job stability and insurance |
|  |  | Job permanence and retirement benefits |
| **Additional Benefits** | Health and Education Support | Comprehensive health insurance |
|  |  | Coverage of children's education costs |
|  | Social and Morale | Social gatherings for employees |
|  |  | Support for employee morale and mental health |
|  |  | Psychological support and fair treatment |
| **Miscellaneous** | Organisationnel improvements | Continuous support and adherence to official decisions |
|  |  | Addressing internal hospital issues to ensure job stability |
|  |  | Alignment with state support and regulations for medical sector sustainability |
|  |  | Fairness in job opportunities and promotions |
